# Determinants of the quality of care relationships in long-term care - a systematic review

**DOI:** 10.1186/s12913-018-3704-7

**Published:** 2018-11-28

**Authors:** Aukelien Scheffelaar, Nanne Bos, Michelle Hendriks, Sandra van Dulmen, Katrien Luijkx

**Affiliations:** 1 Nivel (Netherlands Institute for Health Services Research), PO Box 1568, 3500 BN Utrecht, The Netherlands; 2 Radboud university medical center, Radboud Institute for Health Sciences, Department of Primary and Community Care, Nijmegen, The Netherlands; 3 Reinier van Arkel, Den Bosch, The Netherlands; 4 Faculty of Health and Social Sciences, University of South-Eastern Norway, Drammen, Norway; 5 Tilburg School of Social and Behavioral Sciences, Tranzo, Scientific Center for Care and Welfare, Tilburg University, Tilburg, The Netherlands

**Keywords:** Care relationship, Client-professional relationship, Quality of care, Determinants, Client perspective, Professional perspective, Long-term care

## Abstract

**Background:**

The quality of a care relationship between a client and a care professional is seen as fundamental if high-quality care is to be delivered. This study reviews studies about the determinants of the quality of the client-professional relationship in long-term care.

**Methods:**

A systematic review was performed using the electronic databases of Medline, Psycinfo, CINAHL and Embase. The review focused on three client groups receiving long-term care: physically or mentally frail elderly, people with mental health problems and people with physical or intellectual disabilities. Included studies concern clients receiving inpatient or outpatient care and care professionals who provided recurring physical and supporting care for a long period of time. The studies we included contained primary empirical data, were written in English and were published in peer-reviewed journals. Data extraction was carried out by two researchers independently.

**Results:**

Thirty-two studies out of 11,339 initial hits met the inclusion criteria. In total, 27 determinants were revealed, six at the client level, twelve at the professional level, six between the client and care professional levels and three at the contextual level. The data analysis showed that most determinants were relevant in more than one client group.

**Conclusions:**

This is the first review that looked at determinants of the quality of the care relationship for three large client groups receiving long-term care. It suggests that the current client group-specific focus in research and quality improvement initiatives for care relationships might not be needed. Care organisations can use the findings of this review as guidance on determinants to look for when mapping the quality of a care relationship in order to get a picture of specific points of attention for quality improvement.

## Background

Care relationships between clients and care professionals have received considerable attention in research in recent years. Worldwide, there is a drive to redress the imbalance in care from an ethos that is medically dominated, disease orientated and often fragmented to one that focuses on relationships and people [1]. Relationships are perhaps the most visible feature of the enactment of person-centred care [2]. Especially in long-term care, relationships between clients and care professionals are seen as a fundamental determinant for providing high-quality care, because these relationships are maintained for long periods of time [3]. Long-term care consists of ‘a range of services and assistance for people who, as a result of mental and/or physical frailty and/or disability over an extended period of time, depend on help with daily living activities and/or are in need of permanent nursing care.’ [4] Furthermore, the variable and fluctuating nature of care relationships makes care relationship experiences very singular [5].

Theories focusing on relationships in general are based on several assumptions [6]. Firstly, relationships are never static, but continually changing, growing, re-examined and reinterpreted by both the actors in the relationship and outsiders. Secondly, relationships are inextricably bound with social interactions: relationships are created primarily through social interaction between two people. Thirdly, each relationship must be examined within its cultural context and the overall patterns of other relationships. Finally, the self and the other party plus the relationship between the two are inextricably bound, not fully separable and influencing each other [6].

Determinants of the quality of a care relationship can be distinguished at four levels: 1) client; 2) care professional; 3) interaction between client and care professional, and 4) context. These levels can be illustrated by the following examples of determinants. At the client level, open attitudes from clients towards care professionals influences care relationships positively [7]. At the professional level, the listening skills of a professional and tailoring the provision of care to the individual needs have a positive effect [8]. At the level of the interaction, reciprocity comes to the fore [9]. Concerning context, lack of time and the workloads of care professionals have been suggested as negatively influencing the development and maintenance of a care relationship [10].

In spite of the available studies carried out on this topic, there is as yet no systematic overview of determinants influencing the quality of client-care professional relationships in long-term care. Moreover, nothing is known about the differences or commonalities between client groups in long-term care. Previous research has focused on specific client groups: older adults who are physically or mentally frail, people with mental health problems or with physical and/or intellectual disabilities. In the Netherlands, these three client groups are the largest groups in long-term care. It is unclear to what extent empirical findings support the focus on one specific client group when studying the client-care professional relationship in long-term care.

This systematic review provides an overview of determinants of the quality of the care relationship in long-term care. The main question in this paper is: *What are determinants of a client-care professional relationship in long-term care according to clients and care professionals?* In answering this question, we will examine the similarities and differences in determinants between different client groups of long-term care. Because high-quality relationships between clients and professionals are a fundamental element of the quality of care, the findings of this review can provide input for quality improvement initiatives for long-term care relationships. This systematic review is part of a larger study that focuses on improving the existing qualitative instruments for monitoring the quality of the care relationship between a client and a care professional. Determining the quality of individual care relationships can show care professionals ways of improving their working processes, which can help improve performance [11].

## Methods

### Study design

To examine what is known about the determinants of the quality of care relationships in long-term care, a systematic review was performed using the electronic databases of Medline, Psycinfo, CINAHL and Embase.

### Search strategy

Search strategies were developed for each database with the assistance of an experienced librarian. So that we could focus on recent evidence, we searched for studies published since 2006. Broad strategies were chosen in order to include as many relevant articles as possible. The search strategies included terms identifying client-professional relationships, long-term care and quality. Only EU countries and non-EU G7 countries were included to ascertain the inclusion of those countries that most likely have similarities in the organisational features in the care provision system. See Table 1 for the search string used in Embase. The date of the last search was 6 August 2018.

**Table 1:** Search strings used in Embase, which was adapted to other databases

Professional-patient relationship	
Mesh 1. doctor patient relation/ or nurse patient relationship/ .ti,ab, kw 2. (professional* adj3 (famil* or client* or patient? or resident?) adj5 (relation* or communicat* or interacti*)).ti,ab. 3. (professional* adj3 (famil* or client* or patient? or resident?) adj5 (relation* or communicat* or interacti*)).kw. 4. (nurse* adj3 (famil* or client* or patient or resident*) adj5 (relation* or communicat* or interacti*)).ti,ab. 5. (nurse* adj3 (famil* or client* or patient or resident*) adj5 (relation* or communicat* or interacti*)).kw. 6. (doctor? adj3 (famil* or client* or patient or resident*) adj5 (relation* or communicat* or interacti*)).ti,ab. 7. (doctor? adj3 (famil* or client* or patient or resident*) adj5 (relation* or communicat* or interacti*)).kw 8. (physician? adj3 (famil* or client* or patient or resident*) adj5 (relation* or communicat* or interacti*)).ti,ab. 9. (physician? adj3 (famil* or client* or patient or resident*) adj5 (relation* or communicat* or interacti*)).kw 10. (staff* adj3 (famil* or client* or patient? or resident?) adj5 (relation* or communicat* or interacti*)).ti,ab. 11. (staff* adj3 (famil* or client* or patient? or resident?) adj5 (relation* or communicat* or interacti*)).kw 12. (care provider* adj3 (famil* or client* or patient? or resident?) adj5 (relation* or communicat* or interact*)).ti,ab. 13. (care provider* adj3 (famil* or client* or patient? or resident?) adj5 (relation* or communicat* or interact*)).kw. 14. (nursing adj3 staff adj5 (communicat* or interact* or behav*) adj15 (famil* or client* or patient or resident*)).ti,ab. 15. (nursing adj3 staff adj5 (communicat* or interact* or behav*) adj15 (famil* or client* or patient or resident*)).kw. 16. interpersonal communication/ 17 ((professional* or nurse* or doctor* or physician* or staff or care provider* or care worker*) adj7 (client? or patient? or resident?)).ti,ab. 18. ((professional* or nurse* or doctor* or physician* or staff or care provider* or care worker*) adj7 (client? or patient? or resident?)).kw. 19. 17 or 18 20. 16 and 19 21. or/1–15,20	
AND Long term care	
Mesh 22. elderly care/ or mental health care/ or long term care/ or home care/ or institutional care/ or residential care/ or nursing/ or homes for the aged/ or nursing home/ or psychiatric nursing/ .ti,ab, kw 23. (elderly care or mental health care or long term care or home care or institutional care or residential care or nursing care or nursing home or psychiatric nursing).ti,ab. 24. (elderly care or mental health care or long term care or home care or institutional care or residential care or nursing care or nursing home or psychiatric nursing).kw. 25. (long adj3 term adj5 care*).ti,ab. 26. (long adj3 term adj5 care*).kw 27. ((mental adj5 health* adj5 servi*) or (mental adj5 hygiene* adj5 service*) or (psychiatric adj5 service*)).ti,ab. 28. ((mental adj5 health* adj5 servi*) or (mental adj5 hygiene* adj5 service*) or (psychiatric adj5 service*)).kw. 29. ((health service* for or home* for or care for or service* for) adj5 (aged* or elder* or elderly or senior*)).ti,ab. 30. ((health service* for or home* for or care for or service* for) adj5 (aged* or elder* or elderly or senior*)).kw. 31. ((health service* for or home* for or care for or service* for) adj7 ((person* adj3 disabilit*) or for disable*)).ti,ab. 32. ((health service* for or home* for or care for or service* for) adj7 ((person* adj3 disabilit*) or for disable*)).kw 33. (elderly care or mental health care or home care or institutional care or residential care or nursing care).ti,ab. 34. (elderly care or mental health care or home care or institutional care or residential care or nursing care).kw 35. Or/22–34	
AND Quality	
Mesh 36. total quality management/ or quality control/ or exp. health care quality/ 37. Patient satisfaction/ti,ab, kw 38. (Quality of adj5 care).ti,ab. 39. (Quality of adj5 care).kw 40. (quality adj3 (improve* or improvement* or indicat*)).ti,ab. 41. (quality adj3 (improve* or improvement* or indicat*)).kw 42. meaningful.ti,ab. 43. meaningful.kw 44. (person centred or person centered or patient centred or patient centered or client centred or client centered or relationship centred or relationship centered).ti,ab. 45. (person centred or person centered or patient centred or patient centered or client centred or client centered or relationship centred or relationship centered).kw. 46. patient satisfaction.ti,ab. 47. patient satisfaction.kw 48. or/36–47	
49. 21 and 35 and 48 50. Limit 49 to yr. = “2006-current”	

The truncation symbol (*) is used as a substitute for any string of zero or more characters in the search term

The wildcard symbol (?) serves as a substitute for one character or none

Inclusion criteria were:

- The topic focused on determinants of the quality of care relationships between clients and care professionals in long-term care.
- The study looked at one or more of the following adult client groups receiving long-term care: physically or mentally frail older adults, clients with mental health problems and clients with a physical and/or intellectual disability. Studies that were included concern clients receiving permanent care and/or assistance with daily living activities.
- Clients received care from care professionals providing recurring physical and supporting care for a long period of time, such as various types of nurses, care assistants, personal carers and paid caregivers. This could be inpatient or outpatient care.
- The article contains primary empirical data and was published in a peer-reviewed journal.
- The study was carried out in EU-27 countries and/or non-EU G7 countries (USA, Canada, Australia, New Zealand, Japan).
- The article was written in English.

We excluded studies that did not meet the inclusion criteria:

- Topic not relevant: studies were excluded that focused on the working relationships between care professionals, between clients, or between clients and their families.
- Studies that focused on unrelated settings such as short-term and specialist units of hospitals or palliative services or irrelevant patient groups (patients receiving acute or short term care, hospital patients, people under 18, oncology patients, clients receiving palliative care, patients with explicitly physical care needs such as diabetes, and patients with urological disorders). We also excluded studies that focused on clients of primary care if clients of long-term care were not clearly distinguished as a subgroup.
- Study that focused on professions less directly involved in giving recurring physical and supporting care, e.g. psychiatrists, medical specialists, dentists, medical students and general practitioners. Moreover, studies focusing on care provided on a voluntary basis were also excluded.
- Articles that were non-empirical or not peer reviewed; for instance systematic reviews, theoretical or conceptual frameworks, editorials, abstract overviews, dissertations, letters and comments.

### Study selection

All search results were transferred to a reference database (ENDNOTE) and duplicates were removed. Firstly, titles and abstracts of the retrieved papers were screened and assessed by one researcher (AS). References that clearly did not meet the inclusion criteria were excluded, all others were retained for the abstract screening, including those references of which the researcher had some hesitations whether the reference fitted all inclusion criteria. Secondly, the abstracts of included papers were screened by two researchers independently (AS and NB). In cases where the two researchers rated an abstract differently, consensus was reached by discussion between the two researchers and a third reviewer [12] was consulted if necessary to make a final decision. At the start of the abstract screening phase, the five authors reviewed and discussed a selection of 15 abstracts to increase inter-researcher reliability. Thirdly, the full texts of the studies included were assessed by two researchers (AS and NB). In cases where the two researchers rated the full text differently, consensus was reached by discussion and the other authors (MH, KL, SvD) were consulted if necessary to make a final decision. Additionally, the reference lists of included articles and some relevant but excluded dissertations (exclusion criterion d) were screened to identify additional relevant studies.

### Data extraction

Articles meeting all the inclusion criteria were retained for data extraction using a data extraction file that contained the following variables: author, title, year of publication, period of data collection, study population (client group, type of care professionals included, study population size), care setting, whose perspective the study focuses on (client, professional or both), country in which the study was carried out, study type (qualitative, quantitative, mixed method), type of data collection (open, semi-structured or structured interviews, observations, focus groups, questionnaires), aim of study, definition of the care relationship, journal, abstract, main results.

### Quality assessment

Two researchers (AS and MH or NB) independently rated the quality of the included studies, using the Mixed Methods Assessment Tool [MMAT] [13]. This tool has been designed for reviewing mixed studies. For each type of study (qualitative and quantitative studies), four items were used to assess the quality. For each item, response categories were ‘yes’, ‘no’ or ‘can’t tell’. Each study received a score ranging from one star (25% of the criteria were met) to four stars (all the criteria were met). For mixed-method studies, three additional mixed-method items were assessed on top of the four items concerning the qualitative part and the four items concerning the quantitative part. No study was excluded on the basis of the quality assessment because we were interested in collecting all possible determinants that have been identified as important for the care relationship.

### Data synthesis

The results were analysed and categorized by the first two authors (AS and NB) independently in the qualitative data analysis program MAXQDA. First, AS and NB separately explored the available determinants in the included manuscripts. This was comparable to an open coding phase in qualitative research: the exploration of included articles provided a long list with determinants. AS and NB made a categorization and subdivision of the determinants in two meetings. This categorization was discussed with all authors. Thereafter, all articles were ‘coded’ using the created list of determinants. Two-thirds of the articles were coded double by two researchers (AS and NB or MH). Next, differences and doubtful cases were compared and discussed until consensus was reached. The remaining articles were coded by one researcher. All determinants had to have been noted in at least one high-quality study (i.e. that met at least 75% of the quality criteria of the Mixed Methods Assessment Tool) in order to be included in the results section**.**

## Results

### Study selection

**Fig. 1:**
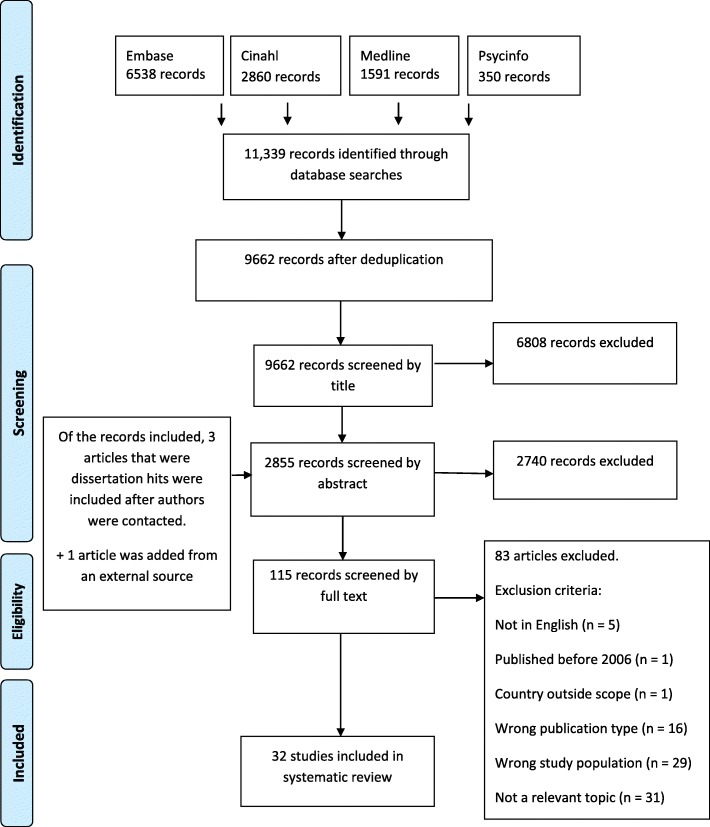
Flow chart of the numbers of articles retrieved and excluded at each stage

The numbers of articles retrieved from the databases that were screened by title, abstract and full text are shown in Fig. 1. The searches resulted in 9662 unique titles. A total of 32 studies were included eventually. A summary of the study characteristics and main results of these 32 studies can be found in Table 2.

**Table 2:** Summary of studies included

Authors (year)	Aim	Data collection	Study population	Perspective 1	Client group 2	Determinants described (level)
Abma et al. (2009)	To understand tensions in the care relationship between professional and client due to different expectations from a care ethics perspective.	open interviews	3 women with multiple sclerosis, chosen out of 15 case studies. The women were receiving care in both inpatient and outpatient settings and reported their experiences with these settings. One case study about a client Jane living in a nursing home was relevant for our study.	C	D	Client: - Attitude (acceptance of situation & complaining or not) Professional: - Attitude (respectful) - Encouragement (encouraging autonomy of client) - Focus on individual client - Take time - Professional competences (technical competences) Between client and professional: - Equality Contextual: - Time
Ahlström & Wadensten (2009)	To explore the encounters in greater depth in close care relationships between personal assistants and disabled people of working age, as well as the prerequisites for and obstacles to the success of such encounters.	open interviews	32 personal assistants who worked for 32 people with serious neurological diseases living at home.	P	D	Client: - Professional: - Attitude (respectful) - Availability (flexibility)- Encouragement (helpful, being positive &encouraging autonomy of client) - Extra effort (take initiative) - Focus on individual client - Take time Between client and professional: - Closeness versus professional distance (professional attitude & informal relationship or friendship) - Reciprocity (personal chemistry) - Trust Other: - Family
Andersen & Spiers (2016)	Exploring the complexities of care, working environments, and knowledge, skills, and efforts of care aides who work in nursing homes.	individual or paired interviews	22 care aides caring for nursing homes residents	P	O	Client: - Abilities - Attitude (open to professional) - Emotional state - Previous life experience Professional: - Attitude (open to client) - Emotional investment or caring - Extra effort (doing more than expected) - Focus on individual client - Professional competences (experience and timing) - Take time - Working in a team Between client and professional: - Continuity (development of relationship) Context: - Hierarchy - Time (workload) Other: - Family
Bäck-Pettersson et al. (2014)	To describe patients’ experiences of supportive conversation as long-term treatment in a psychiatric outpatient context.	focus group interview	6 female patients receiving mental healthcare in an outpatient setting for more than 2.5 years. Clients with: emotional unstable personality disorder (*n* = 1), severe depression, without symptoms of psychosis (*n* = 1), dysthymia (*n* = 1), general anxiety disorder (*n* = 1), and bipolar disorder (*n* = 2). The contact was mostly a qualified nurse.	C	M	Client: - Ask for help Professional: - Attitude (respectful) - Availability - Dependable (confidentiality) Encouragement (creating roles outside of being patient and being helpful) - Extra effort - Focus on individual client - Listen - Professional competences (technical competences) Between client and professional: - Closeness versus professional distance (informal relationship or friendship) - Continuity (development of the relationship) - Equality (collaboration) - Social interaction (open communication)
Bangerter et al. (2016)	Exploring the specific way that a person defines eight key care preferences.	Individual interviews	337 Cognitively capable nursing home residents recruited from 35 Nursing Homes.	C	O	Client: - Professional: - Attitude (friendly and kind, respectful, etiquettes) - Emotional investment or caring - Focus on individual client (interest, genuine concern for client) - Listen - Professional competences (communication) - Take time Between client and professional: - Closeness versus professional distance (professional attitude) - Equality - Reciprocity - Social interaction (use of touch) Context: -
Berggren & Gunnarsson (2010)	To describe the characteristics of the Swedish Personligt Ombud (personal ombudsman; PO) through actual experiences of the service users and more particularly what they find to be significant and helpful features of their relationships with their PO.	open-ended interviews	23 clients with severe mental health impairment. They all had extensive experience of psychiatric care and social services, none of the participants had been admitted to hospital care during the past two years. These clients received short-term individual support from a PO in an outpatient setting.	C	M	Client: - Client in lead Professional: - Attitude (non-judgemental) - Availability - Encouragement (helpful or being supportive, encouraging autonomy of client) - Extra effort (doing more than expected) - Focus on individual client - Professional competences Between client and professional: - Closeness versus professional distance (informal relationship or friendship, professional boundaries, professional distance, being part of a community) - Equality - Reciprocity Contextual: -
Bourgeault et al. (2010)	To examine the role of immigrant care workers in the home and long-term care sectors in Canada, with a particular focus on the relationships with older adults and the implications for quality of care.	semi-structured interviews	77 immigrant care workers, 24 employers, 29 current and future care recipients (older adults) living in nursing home or at home.	C & P	O	Client: - Attitude (open to professional) Professional: - Availability (flexibility) - Dependable - Emotional investment or caring - Focus on individual client - Listen - Professional competences (aware of cultural differences, technical competences) - Take time - Working in a team. Between client and professional: - Closeness versus professional distance (informal relationship or friendship, professional relationship) - Social interaction (language barriers) Contextual: - Setting - Time (workload, lack of backup)
Broer, et al. (2010)	To explore how mental healthcare professionals initiate, improve, and maintain client autonomy while improving other aspects of quality of care.	observations from conferences about projects of care professionals and interviews	Care professionals providing long-term mental healthcare to clients (unspecified amount and without further specification of occupation)	P	M	Client: - Professional: - Availability - Encouragement (being positive, creating roles outside of being patient, encouraging autonomy of client) - Focus on individual client Between client and professional: - Closeness versus professional distance (informal relationship or friendship) - Equality (collaboration) Contextual: -
Cook & Brown-Wilson (2010)	To describe residents’ narratives of their experiences of interacting with staff and making suggestions for practice development.	Observations and interviews	This article draws on two studies: one included 53 older people, the other one included 16 older nursing home residents, 25 staff and 18 family members.	C, P & F	O	Client: - Abilities - Attitude (open to professional) - Client in lead - Previous life experiences Professional: - Attitude (respectful) - Dependable - Extra effort - Focus on individual client - Task-centered - Take time Between client and professional: Closeness versus professional distance (informal relationship or friendship, being part of a community) - Continuity (development of relationship, perceiving stability in the relationship) - Reciprocity - Social interaction (open communication) - Trust Contextual: - Time
Day et al. *(2017)*	To explore consumer concerns, issues and preferences relating to HCPs before the introduction of CDC on the 1 July 2015.	in-depth interviews	Five older people, four women and one man, aged 81 to 90 years participated in the study. Of the five participants, four lived alone and one lived with family. Four participants were receiving HCP services and one was on the waiting list for services.	C	O	Client: - Client in lead Professional: - Focus on individual client (interest, genuine concern for client) - Listen - Take time - Professional competences (communication) Between client and professional: Closeness versus professional distance (informal relationship or friendship) - Continuity (development of relationship) - Reciprocity - Trust Contextual: - Time (workload)
Denhov & Topor (2012)	Exploring the components of helping relationships and the characteristics of helping professionals as described by users who are in various stages of recovery while still undergoing some form of psychiatric care.	open interviews	71 severely mentally ill users of psychiatric care in Sweden, receiving care in both inpatient and outpatient settings. Clients received care from psychotherapists, doctors, social workers or case managers.	C	M	Client: - Professional: - Take time - Attitude (friendly and kind, non-judgemental) Availability - Dependable - Emotional investment or caring - Encouragement (being supportive) - Extra effort (doing more than expected) - Focus on individual client - Listen - Professional competences (timing) Between client and professional: - Closeness versus professional distance (informal relationship or friendship, professional boundaries) - Continuity (development of relationship) - Continuity (perceiving stability in the relationship) - Reciprocity (personal chemistry) - Social interaction - Trust Contextual: -
Dziopa & Ahern (2009)	To explore the attributes of a therapeutic relationship in mental health nursing to determine if there are different ways mental health nurses develop therapeutic relationships.	interviews and Q-sorting	6 mental health nurses were interviewed, thereafter 10 inpatient mental health nurses completed the Q-sorting assignment.	P	M	Client: - Professional: - Attitude (non-judgemental, open to client, respectful) - Encouragement (helpful) - Focus on individual client - Professional competences Between client and professional: - Closeness versus professional distance (professional boundaries) - Continuity - Equality - Social interaction (open communication) - Trust Contextual: -
Eriksen, et al. (2013)	To reveal and express knowledge about the meanings of recognition of clients’ personhood and intrinsic value as human beings, based on mental health workers’ lived experiences of long-term relationships with clients.	multi-stage focus groups	8 mental health workers providing outpatient mental healthcare, all qualified nurses.	P	M	Client: - Professional: - Attitude (non-judgemental, open to client, respectful) - Encouragement - Professional competences Between client and professional: - Closeness versus professional distance (professional distance) Contextual: -
Eriksen et al. (2013)	To describe service users’ understanding of being in relationships with professionals, and how these relationships may limit or enhance recovery.	in-depth interviews	11 people with severe mental illnesses living at home and receiving community-based mental healthcare at least three times a week (unspecified type of care professionals).	C	M	Client: - Abilities - Attitude (open to professional) - Client in lead Professional: - Attitude (non-judgemental) -Availability - Dependable - Encouragement (helpful) - Focus on individual client - Listen Between client and professional: - Equality - Reciprocity (sensing close contact and togetherness) - Social interaction (open communication) Contextual: -
Forsgren et al. (2015)	To explore how nurses experience their everyday interactions with nursing home residents, with a particular focus on interactions with residents with communicative disabilities.	semi-structured interviews	8 nurses, 7 enrolled nurses and 1 nurse’s aide working at six nursing homes and providing care to nursing home residents with communicative disabilities.	P	O	Client: - Abilities (communicative disability) Professional:- Encouragement (being positive) - Focus on individual client - Professional competences – Task-centered Between client and professional: - Continuity (development of relationship) - Equality (power) - Social interaction Contextual: - Time
Huxley et al. (2009)	Exploring what the worker did with and for the user, what the most important actions were, which made the greatest difference, what else could or should be done, and how could the local Support, Time and Recovery [31] service could be improved.	semi-structured interviews	STR workers, mental health service users receiving care in outpatient setting, and managers (unknown number)	C & P	M	Client: - Attitude (open to professional) - Client in lead Professional:- Attitude (non-judgemental, open to client, respectful) - Availability - Dependable (confidentiality) - Encouragement (encouraging autonomy of client) Focus on individual client - Listen - Professional competences (communication) - Take time Between client and professional: - Closeness versus professional distance (informal relationship or friendship, being part of a community -), Continuity (development of relationship) - Reciprocity - Trust Contextual: -
Jones & Moyle (2016)	To explore the nature of relationships in edlerly care services from the perspective of staff.	exploratory interviews	39 direct elderly care staff from 7 residential age care facilities and 12 outpatient community organisations . Respondents were registered nurses, enrolled nurses, personal care workers, community care workers, allied health professionals, and occupational therapists	P	O	Client: - Professional: - Task-centered Between client and professional: - Closeness versus professional distance (professional boundaries) Contextual: - Time Other: - Family
Ljungberg et al. (2017)	to investigate the experience-based knowledge of professionals in outpatient psychiatric services with regard to being personal in their relationships with users.	Open interviews	21 professionals offering treatment in three units for outpatient psychiatric services targeting people with mental health problems who need extensive support. Respondents were 13 mental health care assistants, 4 nurses, 3 psychologists, 1 physiotherapist.	P	M	Client: - Professional: - Focus on individual client (interest, genuine concern for client) - Extra effort (doing more than expected) Between client and professional: - Closeness versus professional distance (informal relationship or friendship, professional boundaries) Contextual: - Setting
McCloughen et al. (2011)	To identify whether consumers and nurses in a mental health rehabilitation setting shared common understandings, attitudes, values, and experiences of nurse–consumer collaboration.	focus groups and a survey for consumers and a survey for nurses.	Consumers of inpatient rehabilitation service of a public psychiatric hospital. The research setting comprised one locked and one open ward and five residential-type complexes. Consumers received less intensive support from nurses and were close to discharge into community accommodation. Three focus group were held with 13 consumers from four residential-type complexes and three focus groups were held with 13 nurses. Thereafter, surveys were completed by 34 nursing staff and 18 consumers.	C & P	M	Client: - Abilities - Attitude (open to professional) - Strategic adapting behaviour Professional: - Attitude (open to client, respectful) - Dependable - Focus on individual client - Listen - Professional competences (communication) - Working in a team Between client and professional: - Equality (collaboration) - Social interaction (open communication) - Hierarchy - Trust Contextual: - Hierarchy - Time (workload, lack of backup)
McGarry (2008)	To provide a clear, in-depth account of the nature of the relationships between nurses and older people within the context of their own home, from the perspective of both nurses and older patients.	semi-structured interviews	13 older patients who were receiving outpatient care from the district nursing service for more than 2 months. 16 community nurses (qualified district nurses, registered nurses and auxiliary nurses)	C & P	O	Client: - Previous life experiences - Attitude (doing as been told) Professional: - Take time - Encouragement (encouraging autonomy of client) Between client and professional: - Closeness versus professional distance (informal relationship or friendship, professional boundaries) - Equality Contextual: - Setting - Time
McGilton & Boscart (2006)	To analyse the perceptions of residents, family members and care providers with regard to close care provider–resident relationships in a LTC setting.	semi-structured interviews	25 residents living in long-term care facilities receiving two or more hours of nursing care per day and their families (or a family member). 32 care providers (registered nurses, licensed practical nurses and healthcare aides).	C, P & F	O	Client: - Abilities - Attitude (not interested, open to professional) Professional: - Attitude (open to client) - Dependable - Extra effort (doing more than expected, take initiative) - Focus on individual client - Listen - Professional competences (technical competences) - Take time Between client and professional: - Closeness versus professional distance (informal relationship or friendship) - Equality (dependency or power) - Reciprocity (personal chemistry) - Social interaction (having a sense of fun or humour, language barriers) Contextual: - Time (workload)
Petriwskyj et al. (2015)	To examine how client engagement is enacted within the context of a large Australian elderly care provider, Blue Care.	interviews and focus groups	85 clients, including 43 clients of community-based services, 32 clients of residential services and 10 clients of retirement living. 94 staff (staff in administration, activities and hospitality, as well as chaplains, assistants in nursing, personal carers, clinical nursing and managers).	C & P	O	Client: - Client in lead - Previous life experiences Professional: - Take time - Emotional investment - Encouragement - Extra effort (doing more than expected) - Focus on individual client - Understand - Professional competences (communication) Between client and professional: - Continuity (development of relationship) - Equality (collaboration) - Equality (dependency, power) - Reciprocity - Trust Contextual: -
Roberts & Bowers (2014)	To develop a conceptual model that explains how residents develop relationships with peers and staff in nursing homes.	unstructured interviews and field observations	15 cognitively intact nursing home residents from 2 nursing homes receiving care from staff (among others nurses)	C	O	Client: - Attitude (acceptance of situation, open to professional) - Previous life experiences - Strategic adapting behaviour Professional: - Attitude (friendly and kind) - Encouragement (encouraging autonomy of client) - Take time Between client and professional: - Closeness versus professional distance (being part of a community) - Reciprocity - Social interaction (having a sense of fun, humour) Contextual: - Time
Rugkåsa, et al. (2014)	To investigate how influencing behaviours were conceptualized by professionals.	structured interviews, in-depth interviews, focus groups	417 patients of community health services took part in structured interviews, 39 patients were additionally interviewed in depth. 48 care professionals (including nurses, psychiatrists, social workers, community support workers, occupational therapists, students and an office manager)	C & P	M	Client: - Professional: - Attitude - Dependable - Encouragement - Extra effort (doing more than expected) - Focus on individual client (interest, genuine concern for client) - Listen - Professional competences (communication) Between client and professional: - Closeness versus professional distance (professional relationship) - Continuity (development of relationship, perceiving stability in the relationship) - Equality (collaboration, dependency, power) - Reciprocity - Trust Contextual: -
Scanlon (2006)	To ascertain the nature and comprehension psychiatric nurses assign to the development of a therapeutic relationship.	semi-structured interviews	Six psychiatric nurses (client group not specified).	P	M	Client: - Professional: - Non-judgemental - Emotional investment or caring - Focus on individual client (interest, genuine concern for client) - Professional competences (communication, timing) - Working in a team Between client and professional: - Closeness versus professional distance (professional boundaries) - Continuity (development of relationship) - Equality (dependency, power) - Reciprocity (sensing close contact and togetherness) - Social interaction (open communication, having a sense of fun or humour) - Trust Contextual: - Time
Schroeder (2012)	To give voice to the lived experiences of older adults with serious mental illness and their perceptions of the healthcare provider relationship	open interviews	8 older adults with a serious mental illness receiving outpatient care from healthcare providers such as a GP, psychiatrist or therapist.	C	O&M	Client: - Professional: - Attitude (Non-judgemental attitude, respectful) - Closeness versus professional distance (being part of a community) - Dependable - Emotional investment (caring) - Encouragement (creating roles outside of being patient, helpful or being supportive, encouraging autonomy of client) - Focus on individual client - Listen Between client and professional: - Closeness versus professional distance (informal relationship or friendship, professional distance) - Continuity (perceiving stability in the relationship) - Equality (dependency, power) - Trust Contextual: -
Sellevold et al. (2013)	To describe healthcare providers’ experience with the ethical challenges and possibilities in the relationship with patients suffering from dementia and their impact on quality care.	in-depth narrative interviews	12 professionals from two different nursing homes providing care to clients suffering from dementia. 4 registered and 8 assistant nurses.	P	O	Client: - Attitude (open to professional) – Emotional state Professional: - Attitude (open to client) – Focus on individual client – Professional competences (communication) Between client and professional: Equality (collaboration) – Reciprocity – Social interaction (use of touch, open communication) - Trust Contextual: -
Shattell et al. (2006)	To examine mentally ill patients’ experiences of what it is like to be understood.	open interviews	20 mentally ill clients. Clients self-identified as having a mental illness.	C	M	Client: - Professional: - Take time – Attitude (non-judgmental, open to client, respectful) – Encouragement – Focus on individual client – Listen – Professional competences (communication) Between client and professional: - Closeness versus professional distance – Continuity (development of relationship) – Equality – Social interaction (use of touch, open interaction) Contextual: -
Shattell et al. (2007)	To describe mental health service recipients’ experience of the therapeutic relationship.	open interviews	20 mentally ill clients. Clients self-identified as having a mental illness.	C	M	Client: - Professional: - Attitude (non-judgmental, open to client) – Emotional investment – Encouragement (being positive, helpful) – Extra effort (doing more than expected) - Focus on individual client – Listen – Professional competences (communication) - Take time Between client and professional: - Closeness versus professional distance – Continuity – Equality – Social interaction (use of touch) Contextual: - Time
Westin & Danielson (2007)	To illuminate and interpret the meaning of residents’ experiences of encounters with nurses in nursing homes.	open interviews	12 residents from 3 nursing homes receiving care for at least 6 months. These residents received care from nurses.	C	O	Client: - Professional: - Attitude (respectful) – Encouragement (being positive, creating roles outside of being patient – Focus on individual client - Take time Between client and professional: - Closeness versus professional distance (being part of a community) - Social interaction (having a sense of fun, humour, open communication) Contextual: - Time
Brown Wilson (2009)	To help understandi the factors that may be significant in forming relationships in care homes and how this may help the community’s development.	participant observation, focus groups and interviews	Interviews with 16 residents, 25 staff members and 18 family members. 8 focus groups of which 3 were held with residents, 2 with family members and 3 with staff members. 256 h of observation. Many residents of one nursing had a degree of cognitive impairment and residents of another nursing home suffered from enduring mental health problems. Staff were unspecified.	C, P & F	O	Client: - Previous life experiences – Being part of a community Professional: - Attitude (respectful) – Dependable – Focus on individual client – Listen – Task centered – Working in a team Between client and professional: - Closeness versus professional distance (informal relationship or friendship) – Continuity – Reciprocity – Social interaction (having a sense of fun, humour) Contextual: - Hierarchy – Setting – Time (workload) Other: - family
Brown Wilson &. Davies (2009)	How these relationships are developed and the contribution that staff make to this process through the routines of care.	participant observation and interviews with residents	256 h of participant observation and interviews with 10 residents of three nursing homes, 25 staff members and 18 family members. Residents of the first nursing home had complex physical healthcare needs and some were cognitively impaired. Residents of the second nursing home had long-term mental health issues and complex healthcare needs. Residents of the third nursing home had complex health needs including mental health problems. Staff were unspecified.	C, P & F	O	Client: - Previous life experiences Professional: - Encouragement (creating roles outside of being patient) – Extra effort – Focus on individual client – Task centered – Take time Between client and professional: - Continuity (development of relationship) – Closeness versus professional distance (being part of a community) - Reciprocity – Social interaction (having a sense of fun, humour, open communication) Contextual: - Time (workload)

1. Included the perspective of client (C), professionals (P) or family (F)

2. Client group concerned: physically or mentally frail older adults (O), people with mental health problems (M), people with disabilities (D)

Most of the studies included were qualitative studies (*n* = 30), plus two mixed-method studies (*n* = 2). The qualitative studies included several study types: ethnographies (n = 3), phenomenological studies (*n* = 9), studies based on grounded theory (*n* = 3), case studies (*n* = 3), and qualitative descriptions (*n* = 12). Both mixed-method studies had a sequential exploratory design in which the qualitative component was followed by the quantitative component. The studies were carried out in Sweden (*n* = 7), the United Kingdom (*n* = 7), Australia (*n* = 5), the United States (*n* = 5), Norway (*n* = 3), Canada (*n* = 3), and the Netherlands (*n* = 2). Fifteen studies focused on physically or mentally frail older people, fourteen on people with mental health problems and two on adults with disabilities. The study population of one study concerned older adults with serious mentally illness, and is within the scope of both older adults and people with mental health problems [14]. Of the studies included, twelve focused on the client perspective, eleven on the professional perspective, and nine on both. Furthermore, four studies that focused on both perspectives also included the family perspective, however the family perspective results were not included in this review. Two articles by Shattell are based on one common data sample [8, 15]. Likewise, three articles by Wilson are based partly on one common data sample [16–18]. These study articles were all included in the data extraction because their results sections focused partly on different topics within the results.

### Quality assessment

Of the 30 qualitative studies, ten met all four quality criteria and ten met three of the four quality criteria (See Appendix 1). These studies appeared to be of good quality. Eight studies met two of the four quality criteria, one study met one criterion and one study met no criteria. Of the two mixed-method studies, McCloughen et al. (2011) met four of the eleven quality criteria and Dziopa & Ahern (2009) met five. Sixteen qualitative studies lacked information on the interaction between the researcher and participants, this information was also not included in the qualitative part of either mixed-method study. Six qualitative studies and both mixed-method studies had no information on how the findings relate to the context in which the data was collected.

### Data summary

The determinants of the quality of care relationships were distinguished at four levels: client, care professional, interaction between client and care professional, and contextual. The essence of the key determinants is described below. It appeared that most were mentioned in two or all three client groups. We have therefore discussed the determinants collectively and only specifically reported when a determinant was found in only one client group [7]. For each level, determinants are described in order from the most often mentioned to the least often. The perspectives included for a study were not related to the levels at which determinants were noted. For example, the study by Roberts and Bowers focused on the client perspective and the findings include determinants of all four levels [7] (see Table 2). Interestingly, determinants at the client level were scarcely described in studies in which only a professional perspective was included.

### Client level

The studies included described determinants at the client level less frequently and extensively than determinants at the professional level. Six determinants of the care relationship were found at the client level, of which two (previous life experiences and emotional state) were only described in studies concerning older adults who were physically or mentally frail.

#### Attitude

The studies described various aspects of the desired attitude of clients [3, 7, 10, 18–25]. Care professionals suggested it is easier to develop close relationships with clients who have open attitudes to care professionals: clients who are willing to interact [10], show interest in a close care relationship [10] and act friendly [7, 10, 23]. Some clients have an accepting attitude, or simply do as they are told [3, 7, 24]. Instances of clients who did not show interest [10] or discriminated against care professionals based on the latter’s skin colour [20] were stated as hindering the development of a care relationship.

#### Previous life experiences

The personal history of a client was mentioned in seven studies focusing on physically or mentally frail older adults [7, 16–19, 24, 26]. Care professionals gather details about clients’ lives so that they can recognise their life experiences [19, 26]. When a care professional knows and recognises the importance of the personal history of a client, this can improve the quality of the care relationship.

#### Client in the lead

Some studies described clients who are ‘in the lead’ in terms of their lives or care, meaning that they make their own decisions and take charge in the relationship [18, 21, 22, 26–28]. A care relationship sometimes seems to serve as a safe learning environment for clients for taking the lead [21, 27]. If a client is in the lead, this adds to the quality of a care relationship.

#### Abilities

Clients’ abilities were described as a determinant of care relationships as well [10, 18, 19, 21, 23, 29, 30]. On the one hand, clients’ lack of social interactional skills and communicative disabilities such as hearing, speaking or visual impairments could reduce the possibilities for developing and continuing a good care relationship [10, 18, 21, 29]. On the other hand, clients’ ability to communicate their wishes could add to a good care relationship [19, 30].

#### Strategic adapting behaviour

Two studies described how clients strategically adapted their behaviour to a certain situation or care professional. Residents determined the right amount and timing of active and passive approaches for receiving care according to their wishes. For example, an active approach could involve a client making specific requests, a more passive approach involved clients allowing staff to provide direct care. These clients adapted in order to avoid negative interactions with staff and maintain a good care relationship [7, 23]. As a result, clients use several strategies to improve the quality of a care relationship.

#### Emotional state

Two studies focusing on physically or mentally frail older adults described the emotional state (specifically anger, frustration and anxiety) of clients as a determinant of care relationships [19, 25]. Negative emotions in a client did not necessarily lead to a negative attitude by the care professional. Professionals tried to understand a client’s bad mood by finding the source or reason [25]. The emotional state of clients might hinder or help the quality of a care relationship.

### Professional level

The studies included described determinants at the professional level extensively. We distilled twelve main determinants. The ones described most often, are focus on the individual client, attitude, and encouragement.

#### Focus on the individual client

Focus on an individual client (the person) was seen as a core determinant of a good relationship in a majority of the studies included [3, 8–10, 14–23, 25–38]. Seeing and knowing the individual needs and priorities of clients are essential for responding to those needs at the right time [9, 17, 19, 23, 26, 27, 31, 35]. It can mean for instance that a care professional simply remembers the client’s name and introduces themselves to the client [34], or knocking before entering and using a formal approach such as ‘Mr. [last name]’ [37]. Ideally, care professionals are interested in a client and their views [9, 10, 15, 23, 28–30, 33, 36–38]. Care professionals need to be capable of understanding the situation of the client by feeling and thinking as though they were the other person [14, 21, 31]. This focus on individual clients might result in clients feeling they are treated like human beings instead of numbers [15].

#### Attitude

The studies described various aspects of the desired attitude by care professionals [3, 5, 7–9, 14, 15, 17–19, 21–23, 25, 27, 30, 31, 33–37]. Clients value care professionals with open or non-judgemental attitudes, those who do not have a predetermined image of a client and who hold back their own opinions and prejudices [5, 14, 15, 19, 21, 23, 25, 27, 33–35]. Respect and dignity for clients are also part of a desirable attitude from care professionals, because clients then feel treated as a person who is worth something in their own right [3, 5, 14–16, 18, 22, 30, 31, 34, 36]. Other attitude aspects mentioned are friendliness [7, 33, 37], honesty [8, 15], being easy to talk to [34], etiquettes [37] empathy [8, 9, 15] being realistic [8] and patience [15]. And vice versa: care professionals with unfriendly, disrespectful attitudes were not valued by clients [3, 36] and such attitudes do not add to the quality of a care relationship.

#### Encouragement

The studies described encouragement as a determinant of the care relationship [5, 8, 9, 14, 17, 21, 26, 27, 29–34, 36]. Encouraging care professionals underline the capabilities of a client, not their disabilities [32]. Especially when clients have a negative self-image or have received negative feedback from people in their environment, instilling hope, being a positive force and promoting independence were described as important [5]. Encouragement also involves care professionals being positive and optimistic in contacts with clients [29, 31, 32, 36]. Clients were encouraged by care professionals to explore their possibilities and make a contribution to the environment and others, for example helping the wellbeing of other clients [14, 17, 32]. Moreover, care professionals try to encourage the autonomy of clients [3, 7, 14, 22, 24, 27, 31, 32] by removing constraints on client autonomy [32] and encouraging clients to be independent and to make their own choices [22, 27].

#### Take time

Several studies describe the importance of professionals taking time and spending time with clients [3, 10, 18–20, 22, 24, 28, 31, 33, 37]. Clients felt ignored by care professionals who did not take time to interact with them [10, 26, 17, 36].

#### Listen

Listening skills and a good ear for a client’s problems, feelings and questions are an important quality of professionals [8–10, 14–16, 20–23, 28, 30, 33, 37], e.g. care professionals who asked or checked whether their understanding was in agreement with the client’s view [15]. When care professionals did not listen to clients, this could result in clients feeling ignored [14]. Corresponding, when care professionals listened to clients, this improved the quality of the care relationships.

#### Professional competences

A number of studies described professional competences [3, 5, 8–10, 15, 19, 20, 22, 23, 25–30, 33, 35, 37]. Care professionals’ competences and expertise comprise technical competences [10] and training [20], non-verbal and verbal communications kills [8, 9, 22, 25, 28, 29, 35, 37], timing of actions, codes of conduct and duty of care [35], work experience [19, 26] and cultural competencies [20]. It follows that these competences add to the quality of a care relationship.

#### Availability

Care professionals need to be flexible and available for clients, which means that they are accessible and reachable for clients when they need them [20–22, 27, 30–33].

#### Extra effort

Extra effort by a care professional contributes to the quality of a care relationship. Extra effort means that care professionals were doing extra things for clients that they did not expect [8–10, 17–19, 26, 27, 30, 31, 33, 38]. Taking initiative [10, 31], letting clients feel special [8], surprising a client with a small present [18, 38] and performing extra tasks besides the usual work such as dog-sitting while a client was in inpatient care [26, 38] were mentioned.

#### Dependable

Clients want care professionals who are dependable and can be relied on [9, 10, 14, 16, 18, 20–23, 30, 33]. Unreliable care professionals did not show up at all when they were called or failed to follow up on promises [10, 14, 18]. Confidentiality is also essential; clients said it was important that some private issues told to a care professional should not be disclosed to others [22, 30].

#### Working in a team

Care professionals need to be willing to work in a team. This requires good communication between care professionals. Cooperation in a team is suggested as affecting care relationships positively, as care professionals complement and back up each other’s tasks [16, 19, 20, 23, 35].

#### Emotional investment

Emotional investment and caring are a key characteristic of a care professional. It expresses the importance for care professionals of investing in a client’s well-being, having a unselfish and committed attitude, and showing genuine concern [8, 14, 15, 19, 20, 26, 33, 35, 37].

#### Task centered

Some care professionals of physically or mentally frail older adults were described as solely focusing on routine tasks [16–18, 29, 39]. A task-centered focus was often related to time shortage and high workload; as a result, these care professionals did not have time to talk to clients [39]. Focusing solely on routine tasks might therefore hinder the quality of a care relationship.

### Interaction between client and care professional level

Six determinants were found between clients and care professionals. The studies included described the determinants on this level extensively and frequently. The determinants equality and closeness versus professional distances were described most often.

#### Equality

Equality is a determinant described in the majority of the studies included [3, 8–10, 14–27, 29–37]. Because the client depends on the care professional’s care and assistance, truly equal relationships seem difficult to achieve [10, 26, 29]. Keeping this in mind, interacting in an equal and collaborative manner is valued by clients and (some) care professionals [3, 9, 14, 21, 23, 24, 34]. Examples were care professionals who provided complete information and treatment options [32], treating a client as an equal [14] and valuing clients’ expert knowledge [23].

#### Closeness versus professional distance

The determinant of closeness versus professional distance is described in a substantial number of the studies included [5, 8–10, 14–16, 18, 20, 22, 24, 27, 28, 30–35, 37–39]. Some care professionals struggle with the borderline area between professional distance and a close relationship, others take a clear position one way or the other [20, 31, 34]. Professional distance includes sticking strictly to professional boundaries and keeping an emotional distance [5, 14, 24, 27, 31, 33–35, 37–39]. Closeness is about friendliness, engagement, sharing personal stories, and professional friendship [10, 16, 18, 20, 22, 24, 27, 28, 30–33, 38]. Care relationships were often compared to friendships, yet at the same time differed clearly. The client need not be concerned with the professional’s own problems and the client can lay more on the shoulders of the care professional. The care professional must maintain professional confidentiality, have unending patience, and not be personally involved in the client’s social network [33]. Moreover, close care relationships seem to be beneficial for clients’ feeling of belonging and being part of a community in which they are valued and accepted [7, 16–18, 22, 27, 36]. This feeling of belonging reduces loneliness and isolation of clients [18, 27, 36]. In short, different clients and care professionals may prefer either greater closeness or more professional distance in the care relationship, and matching these preferences can improve the quality of a care relationship.

#### Continuity

Continuity of a care relationship is experienced as important by clients and care professionals, as was described in fourteen studies [8, 9, 14, 15, 17–19, 22, 26, 28–30, 33–35]. Developing a care relationship requires time and a relationship is continually being built and transformed through interpersonal processes in and outside care routines [28, 29]. Policies of rotating staff or changing primary care professionals led to less continuity in care relationships [17, 18, 30]. Some clients felt anxious that a care professional would quit or would not be assigned to them anymore [33].

#### Reciprocity

Reciprocity between a client and care professional improves the quality of care relationships. Reciprocity means mutual togetherness, personal chemistry, emotional engagement and connection in a care relationship [7, 9, 10, 16–18, 21, 22, 25–28, 31, 33, 35, 37]. Possibilities for reciprocity are created when a client is able to do something for the care professional, such as offering them a drink, or when the client and care professional have similar life experiences, for example the experience of being mothers with children of the same age [26, 27].

#### Trust

Trust was described in several studies as a determinant of care relationships [9, 14, 18, 22, 23, 25, 26, 28, 31, 33–35]. Developing mutual trust takes time and fosters continued contact with clients [9]. Trust also involves a tremendous emotional investment [33]. Some clients only accepted care from the care professionals they trusted and refused care from others [25].

#### Social interaction

Several forms of social interaction were described in the studies that were included [7, 8, 10, 15–18, 20, 21, 23, 25, 29, 30, 33–37]. Social interaction means open, two-way communication and an ongoing dialogue [18, 23, 36]. To achieve such social interaction, the ability of care professionals to communicate on the same wavelength as their clients is underlined [30]. Furthermore, having a sense of fun or humour and non-verbal communication skills such as the use of touch were mentioned as components of social interaction [7, 10, 25]. Open two-way social interaction adds to the quality of a care relationship.

### Contextual level

Three determinants were found at the contextual level. The determinant hierarchy was only described in studies concerning physically or mentally frail older adults.

#### Time

Time constraints, workload or work pressure, inadequate staffing and a lack of backup were found to obstruct the development and retention of care relationships [3, 7, 8, 10, 16–20, 23, 24, 28, 29, 35, 36, 39].

#### Setting

Two studies focusing on care for physically or mentally frail older adults made a distinction between a home care setting and inpatient setting. Home care settings are described as giving clients a higher degree of control over their care and more individual undisturbed care time [20, 24]. A study focusing on persons with mental health problems described differences between particular contexts of inpatient locations or units [38]. In this regard, the care setting can help or hinder the quality of a care relationship.

#### Hierarchy

In two studies focusing on care for physically or mentally frail older adults, lack of decision-making authority and the hierarchy of care professionals with different positions were mentioned [19, 23]. For example, a lack of nurses’ decision-making authority is been described as a practical obstacle to working collaboratively with clients [23]. In a third study, the type of leadership in a care organisation was mentioned as determining the type of care relationships (task-oriented, resident-centred or relationship-centred) [16].

## Discussion

The aim of this review was to provide insights into determinants of the quality of care relationships in long-term care. A systematic review design was chosen to identifying, appraising, and synthesizing all relevant studies on this specific topic. In contrary to a scoping review, a systematic review design is characterized by the predefined search strategy and by the fixed inclusion and exclusion criteria that are defined on beforehand [40, 41]. Determinants were categorised at four levels: client, professional, between client and professional, and contextual. Most determinants were described for two or all three client groups of this study, which were physically or mentally frail older adults, clients with mental health problems and clients with physical and/or intellectual disabilities. The most frequently described determinants were found at the care professional level and between clients and care professionals. At the care professional level, these were ‘focus on the individual client’, ‘encouragement by a care professional’ and ‘attitude of the professional’. At the level between clients and care professionals, these were ‘equality’ and ‘closeness versus professional distance’. Four determinants were found solely for the client group of physically or mentally frail older adults. For this client group only, task-centeredness of care professionals, previous life experiences, emotional state of clients and hierarchy on the work floor came to the fore as determinants of the quality of the care relationship.

Studies focusing on people with physical and/or intellectual disabilities were scarce, resulting in fewer findings for the determinants of the quality of care relationships for this client group. Perhaps for this specific client group, more information is covered by ‘grey’ literature including practice-oriented journals for client group-specific professions. A grey literature study could provide a clearer picture of whether this is the case. Furthermore, this review shows that determinants at the client level are studied less often than determinants at the care professional level. One possible explanation for this might be that care professionals are seen as having more responsibility to make efforts for a good care relationship, given their educational background, their choice of a caring profession and their expected expertise. The responsibility of clients in long-term care relationships might be emphasized less often due to expected shortcomings in the abilities of clients regarding their need for care or assistance. Nonetheless, given the importance of equal care relationships and empowerment of clients, it is important to focus on determinants at the client level as well.

To our knowledge, this is the first review that provides an overview of determinants of the care relationship in long-term care for three client groups. Each of the studies included focused on one specific client group. This review shows that a substantial number of determinants apply to more than one client group. At the moment, it is not clear which determinants are specific to client groups and which have just not been studied in all client groups yet. More research is needed on determinants of the quality of care relationships in more than one client group in order to explore the generalizability of determinants of care relationships. This might expand knowledge of determinants that are specific to client groups as well. Moreover, this finding might suggest that the current client group-specific focus in research into care relationships in long-term care is not necessarily needed. This implication and the findings regarding determinants of the quality of care relationships can provide input for quality improvement initiatives for long-term care relationships.

The studies included in our review were mainly qualitative studies, plus two mixed-method studies. The qualitative studies were primarily exploratory and focused on getting a picture of the wide variety of experiences of clients and/or care professionals. One limitation of qualitative studies is that the external validity of findings is often limited, due to the nature of the methods used. It is also not clear which determinants have the most influence on the quality of a care relationship according to clients and care professionals and what are least likely to be met in existing care relationships, as no weightings are assigned. Moreover, we included all studies regardless of the results of the quality assessment because we were interested in collecting all possible determinants that have been identified as important for the client-care professional relationship. Consequently, the results do not reveal which determinants are most important or have most effect on the quality of care relationships. More research is needed to determine priorities for quality improvement. The current review provides care organisations guidance about what determinants they can look for when examining the quality of the care relationship. The mapping will then give care professionals a picture of specific points of attention for quality improvement. Issues that are specific to the care organisation and care relationship could thereby be taken into account.

Some of the determinants of the quality of care relationships reflect current views on relationships in general in western societies or might be interpreted within contemporary developments in healthcare. For example the focus on the individual client in a care relationship can be placed in the trend towards person-centred care and individualisation. Determinants such as equality, closeness versus professional distance, and clients in the lead can be seen in the light of reducing the social distance between clients and care professionals and the authority of care professionals and broad emphasis on equality in societies. ‘Taking time’ and ‘time and workload’ might illustrate the incompatibilities between providing high-quality, individualised care and cost reduction strategies by national governments.

One limitation of this review may be the broad search string on determinants of care relationships. As a result, we might have missed studies that focused on one specific determinant. For example, it is possible that we have missed studies focusing on reciprocity or another determinant, because the care relationship between client and care professional was not explicitly stated and included. Another limitation is that we did not include ‘grey’ literature in this study, even though these sources may also include relevant knowledge about care relationship determinants. Furthermore, the fact that the title screening was carried out by a single researcher could be seen as a limitation of the study selection process. Besides this, although the classification and grouping of the determinants were carried out by two researchers independently, the classification remains to some extent limited to their interpretations. The interrelatedness of determinants should also be taken into account, both in the interpretation of the findings and in future research.

The influence of clients’ families on the client-professional relationship was outside the scope of this review and therefore not included. Future researchers might look at the studies listed and examine whether the clients’ families also have an effect as another level. Furthermore, in some of the studies that were included, broader concepts such as resident-centred and relationship-centred care were described [16, 17]. These concepts were too broad and abstract to be included as determinants in this review. Future research might look for evidence for a more precise connection between these concepts and individual client-professional relationships.

## Conclusions

This systematic review provides an overview of determinants of the quality of the care relationship in long-term care on four levels: client, professional, between client and professional, and contextual. The studies included each focused on one specific client group in long-term care, specifically physically or mentally frail older adults, people with mental health problems or those with physical and/or intellectual disabilities. This review shows that there is a substantial number of determinants that apply to more than one client group. This might suggest that the current client group-specific focus in research concerning care relationships in long-term care is not necessarily needed. This implication and the findings regarding the determinants of quality for care relationships can provide input for quality improvement initiatives for long-term care relationships. Care organisations can use the findings as a guidance for the determinants to look for when mapping the quality of the care relationship, in order to get clear picture of specific points of attention in quality improvement.

## Appendix 1

### Scores on the Mixed Methods Appraisal Tool (MMAT)

**Table 3:** Scores of qualitative studies

Author (year)	Total score (1.1–1.4)	Screening questions		Qualitative questions			
		Are there clear qualitative and quantitative research questions (or objectives*), or a clear mixed methods question?	Does the collected data allow the research question to be addressed?	1.1. Are the sources of qualitative data relevant for addressing the research question?	1.2. Is the process for analysing qualitative data relevant for addressing the research question?	1.3. Is appropriate consideration given to how the findings relate to the context in which the data was collected?	1.4. Is appropriate consideration given to how the findings relate to the researchers’ influence through their interactions with participants?
Abma et al. (2009)	**	Yes	Yes	Can’t tell	Yes	Yes	No
Ahlström & Wadensten (2009)	**	Yes	Yes	Can’t tell	Yes	Yes	No
Andersen & Spiers (2016)	***	Yes	Yes	Yes	Yes	Yes	No
Bäck-Pettersson et al. (2014)	***	Yes	Yes	Can’t tell	yes	yes	Yes
Bangerter et al. (2016)	**	Yes	Yes	Can’t tell	Yes	Yes	No
Berggren & Gunnarsson (2010)	****	Yes	Yes	yes	yes	yes	Yes
Bourgeault et al. (2010)	****	Yes	Yes	Yes	yes	yes	Yes
Broer et al. (2010)	**	Yes	Yes	Can’t tell	Yes	Yes	No
Cook & Brown-Wilson (2010)	–	Yes	Yes	can’t tell	can’t tell	no	No
Day et al. (2017)	****	Yes	Yes	Yes	Yes	Yes	Yes
Denhov & Topor (2012)	***	Yes	Yes	Can’t tell	Yes	Yes	Yes
Eriksen, et al. (2013) “We are All Fellow Human Beings”:	****	yes	yes	Yes	Yes	Yes	Yes
Eriksen et al. (2013) Challenges in relating to mental health professionals	**	yes	yes	Yes	Yes	No	No
Forsgren et al. (2015)	****	Yes	Yes	Yes	Yes	Yes	Yes
Huxley et al. (2009)	***	Yes	Yes	Yes	Yes	Yes	No
Jones & Moyle (2016)	***	Yes	Yes	Yes	Yes	Yes	No
Ljungberg et al. *(2017)*	*	Yes	Yes	Can’t tell	Yes	No	No
McGarry (2008)	***	Yes	Yes	yes	yes	yes	No
McGilton & Boscart (2006)	***	yes	yes	yes	yes	yes	No
Petriwskyj et al. (2015)	****	yes	yes	yes	yes	yes	Yes
Roberts & Bowers (2014)	****	yes	yes	yes	yes	yes	Yes
Rugkåsa, et al. (2014)	***	yes	yes	yes	yes	yes	No
Scanlon (2006)	***	yes	yes	can’t tell	yes	yes	Yes
Schroeder (2012)	****	Yes	Yes	Yes	Yes	Yes	Yes
Sellevold et al. (2013)	****	yes	yes	yes	yes	yes	Yes
Shattell et al. (2006) She Took the Time to Make Sure She Understood:	**	yes	yes	yes	yes	no	No
Shattell et al. (2007) “Take my hand, help me out:”	**	yes	yes	yes	yes	no	No
Westin& Danielson (2007)	****	yes	yes	yes	yes	yes	Yes
Brown Wilson (2009) Developing community in care homes through a relationshipcentred approach.	***	yes	yes	yes	yes	yes	No
Brown Wilson &. Davies (2009) Developing relationships in long term care environments	**	yes	yes	can’t tell	yes	no	Yes

The number of asterisks (*) show the extent to which the quality indicators were met based on the scores for the four qualitative questions, ranging from one star (one criterion was met) to four stars (all criteria were met)

**Table 4:** Scores of mixed-method studies

Author (year)	Total score (1.1–5.3)	Screening questions		Qualitative questions				Quantitative questions				Mixed-method questions		
		Is there a clear mixed methods question?	Does the collected data allow the research question to be addressed?	1.1.	1.2.	1.3.	1.4	4.1	4.2	4.3	4.4	5.1	5.2	5.3
Dziopa & Ahern (2009)	*(36% of total)	no	yes	can’t tell	yes	no	no	can’t tell	can’t tell	can’t tell	yes	yes	yes	No
McCloughen et al. (2011)	*(36% of total)	no	yes	yes	can’t tell	no	no	yes	can’t tell	can’t tell	no	yes	yes	No

The number of asterisks (*) show the extent to which the quality indicators were met based on the scores for the qualitative, quantitative and mixed method questions, ranging from one star (3–4 of the 11 criteria were met) to four stars (all criteria were met)

### MMAT questions

1.1 Are the sources of qualitative data relevant for addressing the research question?

1.2 Is the process for analysing qualitative data relevant for addressing the research question?

1.3 Is appropriate consideration given to how the findings relate to the context in which the data was collected?

1.4 Is appropriate consideration given to how the findings relate to the researchers’ influence, e.g. through their interactions with participants?

4.1 Is the sampling strategy relevant for addressing the quantitative research question?

4.2 Is the sample representative of the population being studied?

4.3 Are measurements appropriate?

4.4 Is there an acceptable response rate (60% or above)?

5.1 Is the mixed-methods research design relevant for addressing the qualitative and quantitative research questions or aspects of the mixed-methods question?

5.2 Is the integration of qualitative and quantitative data relevant for addressing the research question?

5.3 Is appropriate consideration given to the limitations associated with this integration, e.g. the divergence of qualitative and quantitative data?

## References

[1] McCormack B, McCance T. Person-centred nursing: theory and practice: John Wiley & Sons; 2011.

[2] McCormack BD, S. van, Eide, H. Skovdahl, K. Eide, T.. person-Centred healthcare research, vol. first edition: © 2017 john Wiley & sons ltd. Published 2017 by John Wiley & Sons ltd.; 2017.

[3] Abma TA, Oeseburg B, Widdershoven GA, Verkerk M (2009). The quality of caring relationships. Psychology Research & Behavior Management.

[4] Commission spCe. Adequate social protection for long-term care needs in an ageing Society. Luxembourg: european Union; 2014. http://ec.europa.eu/social/publications

[5] Eriksen KA, Arman M, Davidson L, Sundfor B, Karlsson B (2013). "we are all fellow human beings": mental health workers' perspectives of being in relationships with clients in community-based mental health services. Issues in Mental Health Nursing.

[6] Wilmot WW: Relational communication: McGraw-hill humanities, Social Sciences & World Languages; 1995.

[7] Roberts T, Bowers B (2015). How nursing home residents develop relationships with peers and staff: a grounded theory study. Int J Nurs Stud.

[8] Shattell MM, Starr SS, Thomas SP (2007). ‘Take my hand, help me out’: mental health service recipients' experience of the therapeutic relationship. Int J Ment Health Nurs.

[9] Rugkåsa J, Canvin K, Sinclair J, Sulman A, Burns T (2014). Trust, deals and authority: community mental health Professionals' experiences of influencing reluctant patients. Community Ment Health J.

[10] McGilton KS, Boscart VM (2007). Close care provider-resident relationships in long-term care environments. J Clin Nurs.

[11] Berwick DM, James B, Coye MJ (2003). Connections between quality measurement and improvement. Med Care.

[12] Bomhoff M, Paus N, Friele R: Niets te klagen: onderzoek naar uitingen van ongenoegen in verzorgings-en verpleeghuizen. 2013.

[13] Pace R, Pluye P, Bartlett G, Macaulay AC, Salsberg J, Jagosh J, Seller R (2012). Testing the reliability and efficiency of the pilot mixed methods appraisal tool (MMAT) for systematic mixed studies review. Int J Nurs Stud.

[14] Schroeder R (2013). The seriously mentally ill older adult: perceptions of the patient-provider relationship. Perspectives in Psychiatric Care.

[15] Shattell MM, McAllister S, Hogan B, Thomas SP (2006). “She took the time to make sure she understood”: mental health Patients' experiences of being understood. Arch Psychiatr Nurs.

[16] Brown Wilson C (2009). Developing community in care homes through a relationship-centred approach. Health Soc Care Community.

[17] Wilson CB, Davies S (2009). Developing relationships in long term care environments: the contribution of staff. J Clin Nurs.

[18] Cook G, Brown-Wilson C (2010). Care home residents’ experiences of social relationships with staff: the importance of the emotional aspect of caring and how it influences the quality of life of residents and staff is demonstrated by the findings of two studies discussed by Glenda cook and Christine Brown-Wilson. Nursing older people.

[19] Andersen EA, Spiers J (2016). Care Aides' relational practices and caring contributions. J Gerontol Nurs.

[20] Bourgeault IL, Atanackovic J, Rashid A, Parpia R (2010). Relations between immigrant care workers and older persons in home and long-term care. Can J Aging.

[21] Ådnøy Eriksen K, Arman M, Davidson L, Sundfør B, Karlsson B (2013). Challenges in relating to mental health professionals: perspectives of persons with severe mental illness. Int J Ment Health Nurs.

[22] Huxley P, Evans S, Beresford P, Davidson B, King S (2009). The principles and provisions of relationships: findings from an evaluation of support, time and recovery workers in mental health services in England. J Soc Work.

[23] McCloughen A, Gillies D, O'Brien L (2011). Collaboration between mental health consumers and nurses: shared understandings, dissimilar experiences. Int J Ment Health Nurs.

[24] McGarry J (2010). Relationships between nurses and older people within the home: exploring the boundaries of care. Int J Older People Nursing.

[25] Sellevold GS, Egede-Nissen V, Jakobsen R, Sorlie V (2013). Quality care for persons experiencing dementia: the significance of relational ethics. Nurs Ethics.

[26] Petriwskyj A, Gibson A, Webby G (2015). Staff members' negotiation of power in client engagement: analysis of practice within an Australian aged care service. J Aging Stud.

[27] Berggren UJ, Gunnarsson E (2010). User-oriented mental health reform in Sweden: featuring 'professional friendship'. Disability & Society.

[28] Day J, Taylor ACT, Summons P, Van Der Riet P, Hunter S, Maguire J, Dilworth S, Bellchambers H, Jeong S, Haydon G (2017). Home care packages: insights into the experiences of older people leading up to the introduction of consumer directed care in Australia. Aust J Prim Health.

[29] Forsgren E, Skott C, Hartelius L, Saldert C (2016). Communicative barriers and resources in nursing homes from the enrolled nurses' perspective: a qualitative interview study. Int J Nurs Stud.

[30] Back-Pettersson S, Sandersson S, Hermansson E (2014). Patients' experiences of supportive conversation as long-term treatment in a Swedish psychiatric outpatient care context: a phenomenological study. Issues Ment Health Nurs.

[31] Ahlstrom G, Wadensten B (2010). Encounters in close care relations from the perspective of personal assistants working with persons with severe disability. Health Soc Care Community.

[32] Broer T, Nieboer AP, Bal RA (2010). Quest for client autonomy in improving long-term mental health care. Int J Ment Health Nurs.

[33] Denhov A, Topor A (2012). The components of helping relationships with professionals in psychiatry: users' perspective. Int J Soc Psychiatry.

[34] Dziopa F, Ahern K (2009). Three different ways mental health nurses develop quality therapeutic relationships. Issues Ment Health Nurs.

[35] Scanlon A (2006). Psychiatric nurses perceptions of the constituents of the therapeutic relationship: a grounded theory study. J Psychiatr Ment Health Nurs.

[36] Westin L, Danielson E (2007). Encounters in Swedish nursing homes: a hermeneutic study of residents’ experiences. J Adv Nurs.

[37] Bangerter LR, Van Haitsma K, Heid AR, Abbott K (2015). “Make me feel at ease and at home”: differential care preferences of nursing home residents. The Gerontologist.

[38] Ljungberg A, Denhov A, Topor A (2017). A balancing act—how mental health professionals experience being personal in their relationships with service users. Issues Ment Health Nurs.

[39] Jones C, Moyle W (2016). Staff perspectives of relationships in aged care: a qualitative approach. Australas J Ageing.

[40] Peters MD, Godfrey CM, Khalil H, McInerney P, Parker D, Soares CB (2015). Guidance for conducting systematic scoping reviews. Int J Evid Based Healthc.

[41] Arksey H, O'Malley L (2005). Scoping studies: towards a methodological framework. Int J Soc Res Methodol.

