# Correction to: Determinants of the quality of care relationships in long-term care – a systematic review

**DOI:** 10.1186/s12913-019-4304-x

**Published:** 2019-07-08

**Authors:** Aukelien Scheffelaar, Nanne Bos, Michelle Hendriks, Sandra van Dulmen, Katrien Luijkx

**Affiliations:** 1 Nivel (Netherlands Institute for Health Services Research), PO Box 1568, 3500 BN Utrecht, The Netherlands; 2 Department of Primary and Community Care, Radboud university medical center, Radboud Institute for Health Sciences, Nijmegen, The Netherlands; 3 Reinier van Arkel, Den Bosch, The Netherlands; 4 Faculty of Health and Social Sciences, University of South-Eastern Norway, Drammen, Norway; 5 Tilburg School of Social and Behavioral Sciences, Tranzo, Scientific Center for Care and Welfare, Tilburg University, Tilburg, The Netherlands

**Correction to: BMC Health Serv Res (2018) 18:903**

**https://doi.org/10.1186/s12913-018-3704-7**

In the original publication of this article [1], there is a layout mistake in the column “McCloughen et al. (2011)” of Table 2.

The corrected column should be:

**Table:** 

Authors (year)	Aim	Data collection	Study population	Perspective 1	Client group 2	Determinants described (level)
McCloughen et al. (2011)	To identify whether consumers and nurses in a mental health rehabilitation setting shared common understandings, attitudes, values, and experiences of nurse–consumer collaboration.	focus groups and a survey for consumers and a survey for nurses.	Consumers of inpatient rehabilitation service of a public psychiatric hospital. The research setting comprised one locked and one open ward and five residential-type complexes. Consumers received less intensive support from nurses and were close to discharge into community accommodation. Three focus group were held with 13 consumers from four residential-type complexes and three focus groups were held with 13 nurses. Thereafter, surveys were completed by 34 nursing staff and 18 consumers.	C & P	M	Client: - Abilities - Attitude (open to professional) - Strategic adapting behaviour Professional: - Attitude (open to client, respectful) - Dependable - Focus on individual client - Listen - Professional competences (communication) - Working in a team Between client and professional: - Equality (collaboration) - Social interaction (open communication) - Hierarchy - Trust Contextual: - Hierarchy - Time (workload, lack of backup)

The original article has been corrected.
